# New products

**DOI:** 10.1155/S1463924689000477

**Published:** 1989


Journal of Automatic Chemistry Vol. 11, No. 5 (September–October 1989), pp. 240–245

### New products

### New system triples capabilities

Sales of Yokogawa's Micro Excel (MXL) Distributed Control System introduced in Europe in April are accelerating. World sales are now above 600 contracts, achieved in six months of marketing. The company expects continued appreciation from the market for this system's significantly up-rated capabilities.

The MXL system configurations and functions cover five operator stations (32 blt MC69020 processor, distributed real-time BASIC), Field Control Units up to 16 (standard), (16 blt MC68000 CPU), and is capable of handling 256 control loops. The system RL-bus (standard: 1 km, 1 Mbps) is expandable to 15 km with an optical fibre link. (The system has effectively pulled down the capabilities of the supervisory computer into reach of small and medium-sized users over a wide spectrum of industries.) This comprehensive and cost-effective machine has been designed for real-time production control of continuous and batch operations with high expandability. It offers the benefits of a large unit, but its price is that of a small unit. The recent European introduction of MicroExcel will naturally benefit from earlier international operational lessons. MXL has been designed to grow with clients into the next century.

*For further information contact: Yokogawa Electrofact, UK Liaison Office, Grove House, 551 London Road, Isleworth, Middlesex TW7 4DS, UK. Tel.: 01 847 3051.*

### Environmental monitoring service launched

Infomat *Green Alert* brings together the latest information on environmental issues. Derived from over 600 newspapers and trade journals in 12 languages Infomat's *Green Alert* is a digest of key developments in the environmental debate. Topics covered include:

- General developments – new studies, consumer attitudes and trends, global issues, the greenhouse effect, CFCs, PCBs, political implications.
- New EEC and government initiatives.
- Incidents – spillages and other pollution incidents.

Published weekly, *Green Alert* costs £240 p.a.

*For further information contact: Infomat Ltd, Votec Centre, Hambridge Lane, Newbury, Berkshire RG14 5TN. Tel: (0635) 34867.*

### Strip chart recorders offer long-term service

E-5200, from the French company Meci, is a new DIN 144 × 144 strip chart recorder that offers both high functionality and ease of use.

Its modular design means that sub assemblies can be easily accessed for maintenance, while a reinforced plastic housing makes it unusually rugged and ensures an extended operating life.

Designed to take roll or fanfold recording paper, the E-5200 can operate at two chart speeds, either 20 or 60 mm/h, and ×10 multiplier switches can be used with any chart speed option.

There is an option for a second independent measurement channel which can be switched on while recording is in progress.

In the two-channel version, the recorder uses red and blue felt-tip pens capable of covering up to 1500 metres of chart.

Charts can be consulted without interrupting the recorder's operation and it is also possible to monitor recording visually through the front of the casing. The E-5200 relies on a highly accurate potentiometric balance system, giving it an accuracy of 0·5%.

*Details from: Meci, 18 rue Goubet F75019 Paris, France.*

### New automated glucose and L-lactate analyser

The automated YSI 2300 STAT Glucose and L-Lactate analyser adds autocalibration and automatic sample aspiration to the rapid response, whole-blood capability and low cost per test of the present YSI analysers.

The 2300 STAT is designed for laboratories serving surgery, intensive care, neonatal units and for research. Completely automated, the analyser is always calibrated and ready for use.

Using YSI's patented immobilized enzyme technology, the 2300 STAT provides precision and accuracy substantially equal to its predecessors. The unit measures glucose and lactate simultaneously, printing and displaying results in less than 90 s.

The user presents a sample and presses the keypad – the 2300 STAT aspirates 25 μl for analysis. It will determine glucose levels in whole blood, plasma or serum, and lactate levels in whole blood, plasma or cerebrospinal fluid.

The unit can interface with a PC through its RS232C port. The glucose measurement range is 0 to 500 mg/dl; the lactate range is 0 to 135 mg/dl.

*Details from: YSI Incorporated, Yellow Springs, Ohio 45387, USA. Tel.: 513 767-7241.*

### Thermography system

A sophisticated thermography system has been launched by Leicester-based Rank Taylor Hobson. Talytherm has resulted from the company's work with the Ministry of Defence in the military thermal imaging field and has now been developed to suit a wide variety of commercial applications.

The system incorporates a new software package, TEMPS (Thermal Transmission Measurement Processing Software). The software is based on the highly successful Microsoft Windows which make choosing and running programmes easier and more flexible.

The system can scan a scene at full standard TV rates, 50 Hz field rate and 15 KHz infra-red line rate, it has a sensitivity of 0·03 °C detectable temperature difference, and it offers 512 scanned infra red lines with 780 samples per line and image processing in a 512 × 512 image array.

Data can be viewed via a monochrome or full colour monitor or as a hard copy print-out. Data can be frozen or down loaded for future retrieval or for further analysis. The advanced design also allows for real time viewing.

During the development stage of Talytherm, systems have already been sold throughout the world. Applications have included medical and industrial research, non-destructive testing and infra red signature measurement.

*For further information, contact: Merv Davies, Business Manager, Industrial Thermography, Rank Taylor Hobson, PO Box 36, New Star Road, Leicester LE4 7JQ, UK. Tel.: 0533 763771.*

### Expert systems demonstration software

Demonstration software for Philips Analytical's new chromatography enhancer expert system is now available free of charge in the UK. Developed in collaboration with European industrial and academic centres, PU6106 expert system software brings new levels of speed, ease and sophistication to chromatographic parameter optimization.

The IBM PC compatible software selects physical parameters such as flow rate, column dimensions and detector response times for optimum speed of analysis at the required sensitivity. As a result, analysts can develop fast chromatographic methods, optimise for the most complex parameters and ensure that optimal results are always achieved from instrumentation.

*The demonstration disk can be obtained from: Lesley McLay, Philips Analytical Chromatography, York Street, Cambridge CB1 2PX, UK. Tel.: 0223 358866.*

### Remote controller monitoring system for toxic gases

Recently announced by Bruel & Kjaer's Gas Analysis Division is a rugged, but highly sensitive, toxic gas monitor designed for long-term unattended monitoring.

The Type 1306 Toxic Gas Monitor can be used for plant-wide detection of accidental gas release, or monitoring of process emissions, in accordance with environmental protection and occupational health and safety requirements. Running costs are low since the system functions under extreme environmental conditions with minimum attention. Frequent self-test guarantees reliable operation. Recalibration and filter replacement are the only routine maintenance required, typically at three-monthly intervals.

The measurement technique is based on photoacoustic infra-red spectroscopy which offers advantages over existing methods in speed of response, sensitivity and immunity to interferents. The instrument is highly selective by virtue of a wide choice of optical filters, enabling detection of almost any gas which absorbs infra-red light, while compensating for the effects of water vapour. The detection threshold is gas-dependent and in the parts per billion to parts per million region. Wide dynamic range allows measurement of concentrations four orders of magnitude higher than the detection threshold.

Bruel & Kjaer supplies software enabling one to 31 gas monitors to be controlled by an IBM PC or compatible computer. Highly graphic screen displays and simple on-screen selection of commands make the system very easy to operate. The user selects a normal measurement interval, which is supplanted by intensification and alarm modes when preset thresholds are exceeded. When alarm levels are detected, measurements are made continually. Storage is provided for the most recent measurement and self-test sequences, the data being available on request without disrupting operation. Results can be printed or plotted if required.

Applications areas for the new monitors are widespread and include engineering, chemical, pharmaceutical, mining, oil and gas, water, building, and waste disposal industries.

*Further information from: John Bancroft, Bruel & Kjaer (UK) Ltd, 86 East Road, Longsight, Manchester M12 5GY, UK. Tel.: 061 224 7590; fax: 061 248 6140.*

### 'Split version' model 9150/SV CO and $O_2$ and flue gas analyser

Teledyne's Model 9150/Sv helps improve combustion efficiency and reduce emissions by monitoring carbon monoxide (CO) and oxygen ($O_2$) in flue gas. The 9150/SV's simple, low cost on-stack sampling design is ideal for monitoring natural gas-fitted combustion and other clean-burning applications. The 9150/SV features a 'split version' configuration:

The analysis section contains the sampling system and CO and $O_2$ sensors, and mounts on or near the stack-providing fast response and reduced installation costs; the separate control unit contains meter readouts and electronic controls and mounts remotely away from the stack in a control room, instrument shed or other convenient location. The 9150/SV also features three $O_2$ ranges (0/5 0–10 and 0–25% $O_2$) and two CO ranges (0–500 and 0–1000 ppm CO).

Accessories, such as probes and external sample lines, are available. For coal-fired and other dirty applications, preconditioners and optional sampling systems are available. Other options include alarms, mADC signal output and NEMA four purged enclosures.

*More information from: Teledyne, The Harlequin Centre, Southall Lane, Southall, Middlesex UB2 5NH, UK. Tel.: 01 571 9596; fax: 01 571 9439.*

### Autobomb systems

The latest marque Autobomb is an improved version of the Gallenkamp adiabatic calorimeter. It has many new features designed to improve performance and safety and it forms the basis of the new Gallenkamp Calorimetry Systems. Three systems are available, the choice of system depends upon the level of sample throughput and the type of data handling required.

*System AC*

The Autobomb plus the new Gallenkamp AutoCal enables fully automatic microprocessor control of the test procedure. Catering for most applications, the AutoCal can control two Autobombs and automates the loading of weights, firing the bomb, temperature monitoring and recording, and the calculation of gross calorific value. Corrections can be added to obtain net calorific value. The AutoCal can store data and a built-in printer will give a hard copy of the results.

*System AT*

Designed for situations where full automation is not required, the System AT offers digital electronic thermometry in place of the AutoCal. The Gallenkamp AutoTherm gives a clear easy to read display of temperatures in °C or K to three decimal places. It uses the same temperature probes as the AutoCal. The firing of the bomb and all calculations are carried out manually. The AutoTherm can monitor the temperature rise in two bombs by manually switching between thermometer channels.

*System MT*

Where low cost is of prime importance this sytem offers the standard Autobomb console together with mercury-in-glass calorimetry thermometers.

*Details from: Gallenkamp, Belton Road West, Loughborough, Leicestershire LE11 0TR, UK. Tel.: 0509 237371.*

### Difficult analysis solved by refractometer system

Direct concentration measurements of emulsions and suspensions, such as PVA glue, chocolate sauce, salad dressing and any of the many products containing gelatin, has always been difficult. However, a refractometer system developed by Index Instruments has overcome most of these problems.

The GPR11-37 Refractometer features an emulsion measuring scale which allows all of these difficult analyses to be carried out simply, quickly and accurately. The GPR11-37 is especially suited to measuring milky or volatile substances.

A thin layer of sample, which can be liquid or set is applied directly to the instrument's sapphire prism and the cover closed. Results accurate to within 0·0001% are displayed on the digital readout in seconds. After the analysis, the sample is simply wiped clean from the sample window surface.

The measurement range is Nd 1·32 to Nd 1·70. Calibration is simple and there are a wide variety of sample cells available for continuous flow-through analysis as part of a process control system.

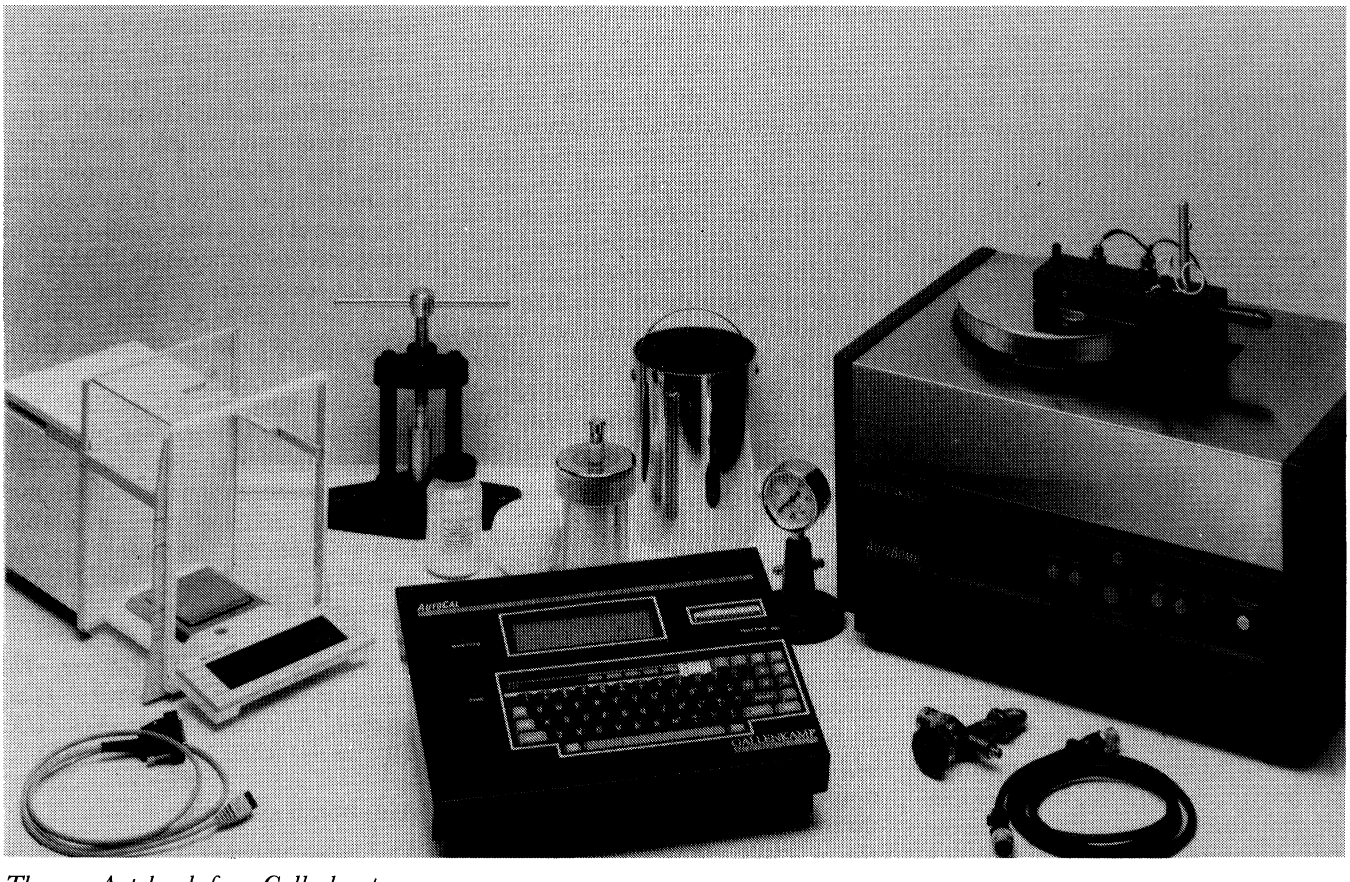

*The new Autobomb from Gallenkamp.*

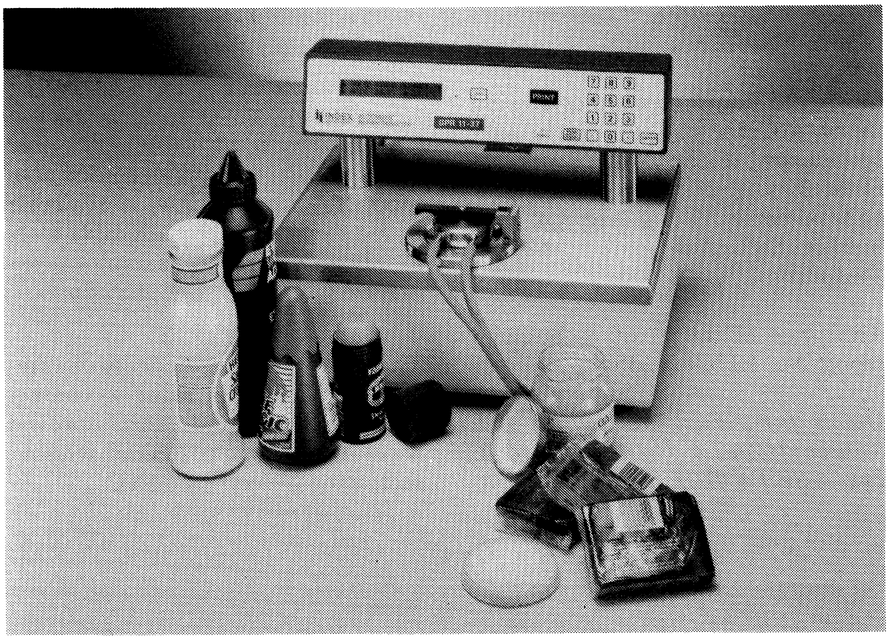

*Direct concentration measurements on difficult samples such as emulsions, suspensions and gelatin bearing products, can now be made directly with the new GPR11-37 Refractometer.*

Proven applications for the GPR11-37 system include gelatin solutions for products as diverse as table jelly and stick deodorant. One manufacturer producing 30 000 tonnes of gelatin a year uses the GPR11-37 extensively.

*For further information or applications advice on the use of refractometers for measuring emulsions and suspensions contact: Index Instruments Ltd, Bury Road Industrial Estate, Ramsey, Huntingdon PE17 1NA, UK. Tel.: 0487 814313.*

### DNA sequencing software updated

Sequences can be accessed by author or keyword, and full annotations are available for any sequence using the CD-ROM laser disk version of 'Gen-Bank' and NBRF-PIR data banks. These features are offered with the new MicroGenie verison 6.0 software from Beckman.

This software features higher resolution graphs on all IBM PS/2 computers, the ability to fit more sites around circular restriction maps, and the ability to construct linear restriction maps with sites labelled along a single line.

This update provides an additional method for calculating hydrophobicity of proteins, allows calculation of the hydrophobic moment of alpha helical protein regions, and is compatible with a Hewlett Packard LaserJet printer.

*Details from: Beckman, Progress Road, Sands Industrial Estate, High Wycombe, Buckinghamshire, UK. Tel.: 0494 441181.*

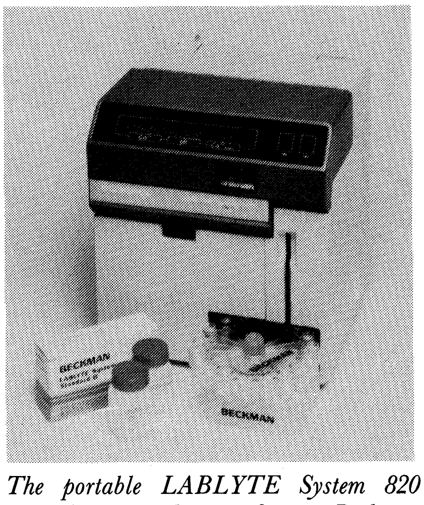

*The portable LABLYTE System 820 electrolyte analyser from Beckman Instruments provides critical care analysis for ionized calcium. The instrument is an economical way to acquire Ionized Calcium and Hydrogen-ion activity (pH) capability when analysing for potassium (K) and sodium (Na). It features ion-selective electrode technology and is designed for STAT response by providing whole blood analysis capability. Details from Beckman Instruments Inc., 2500 Harbor Blvd., Fullerton, California 92634-3100, USA.*

### Electrolyte system offers STAT-interrupt and instrument chemistry expandability

Beckman Instruments has introduced an expandable SYNCHRON EL-ISE$^{TM}$ electrolyte system, which was to be a stand-alone primary routine electrolyte analyser or a complement to a random access profiler. It is available as an automated two-channel analyser for Na, K, Cl, and $CO_2$ analyses. Four operation modes – auto, manual, batch and STAT – are available with each system. STAT-interrupt with results in less than a minute and instrument chemistry expandability in just a few hours are key features of the SYNCHRON EL-ISE.

The high-throughput analyser completes more than 100 total electrolyte panels per hour, and features a 40-sample turntable for high-volume testing. Sample requirements are only 50 μl for serum, plasma or CSF, and 60 μl for urine.

Available in six languages – English, French, German, Italian, Spanish and Japanese – software with every SYNCHRON EL-ISE provides capability for a patient sample identification number for each specimen. Other features include up to six operator-defined sample comments such as 'lipaemic' or 'possible hepatitis'; storage and calculation of QC statistics for up to nine controls; preprogramming for up to six panels; a selection of two Anion Gap formulas; memory for storage and recall of up to 120 samples from three trays; LCD display; and an INTERLINK interface to other SYNCHRON clinical systems.

*For more information, contact: Beckman Instruments, Inc., 2500 Harbor Blvd., Fullerton, California 92634-3100, USA.*

### Spain seen as hot spot in Europe's automated clinical chemistry analyser market

Spain will be the hot spot in the largely mature European market for automated clinical chemistry analysers over the next five years, according to a new study researched and written in Europe by Frost and Sullivan Ltd.

Spanish consumption will jump 61% from \$8·4 million in 1988 to \$13·5 million in 1993 while total regional sales increase 15% from \$158·1 million to \$181·7 million.

The number of new units sold in Europe each year is expected to increase by 2% from 1226 in 1988 to 1253 in 1993. Due to their greater throughput, the European installed base will fall steadily through the forecast period from approximately 12 000 to 10 600.

France, which has more small- and medium-sized private clinical laboratories than other countries, is the largest market for automated clinical chemistry analysers. Its 1989 sales of \$41·2 million are expected to grow 10% to \$45·5 million in 1993. The West German market should expand at the same rate from \$38·6 million to \$42·6 million. Italy's consumption is forecast to grow 16% from \$35 million to \$40·6 million over the period, while the UK market increases 14% from \$20·4 million (£11·4) to \$23·3 million (£13 million).

Frost & Sullivan believes that the main demand over the next five years will be for discrete analysers which offer random access to a wide range of tests, the capacity to incorporate new tests, and the ability to perform stat analyses with minimal interruption to routine analysis.

The market for centrifugal analysers is expected to decline, but 'capsule' chemistry may increase its market share if the instruments can demonstrate their functionality. Spectrophotometric methods of analysis should continue, and perhaps increase, their domination of the market as spectrophotometric methods for the analysis of electrolytes are developed and accepted.

The most important technology developments are expected in the areas of data handling and computer control, bar-coding of samples and test selection, and the ability to handle the primary sample tube.

The movement toward decentralization testing will be much slower in Europe than in the US, due largely to the level of government control over the provision of health care services in most European countries.

Of the 28 major companies serving the European market, Boehringer Mannheim is the leader with its Hitachi analysers accounting for approximately a quarter of the market. Second in market share is Technicon.

The price of the Frost & Sullivan report No. E1169 is \$3500.

*Details from: Frost and Sullivan Ltd, Sullivan House, 4 Grosvenor Gardens, London SW1W 0DH. Tel.: 01 730 3438.*

### Robot offers fully integrated laboratory automation

A laboratory robot for automating virtually any liquid handling task has been developed by Kemble Instruments. The polar co-ordinate robot (the Autolab 500) is available with a range of interchangeable tools to allow control of sample preparation or entire assay procedures.

The robot offers the benefits of automation, including reduced contact with hazardous fluids, and improved accuracy and consistency, without sacrificing processing speed.

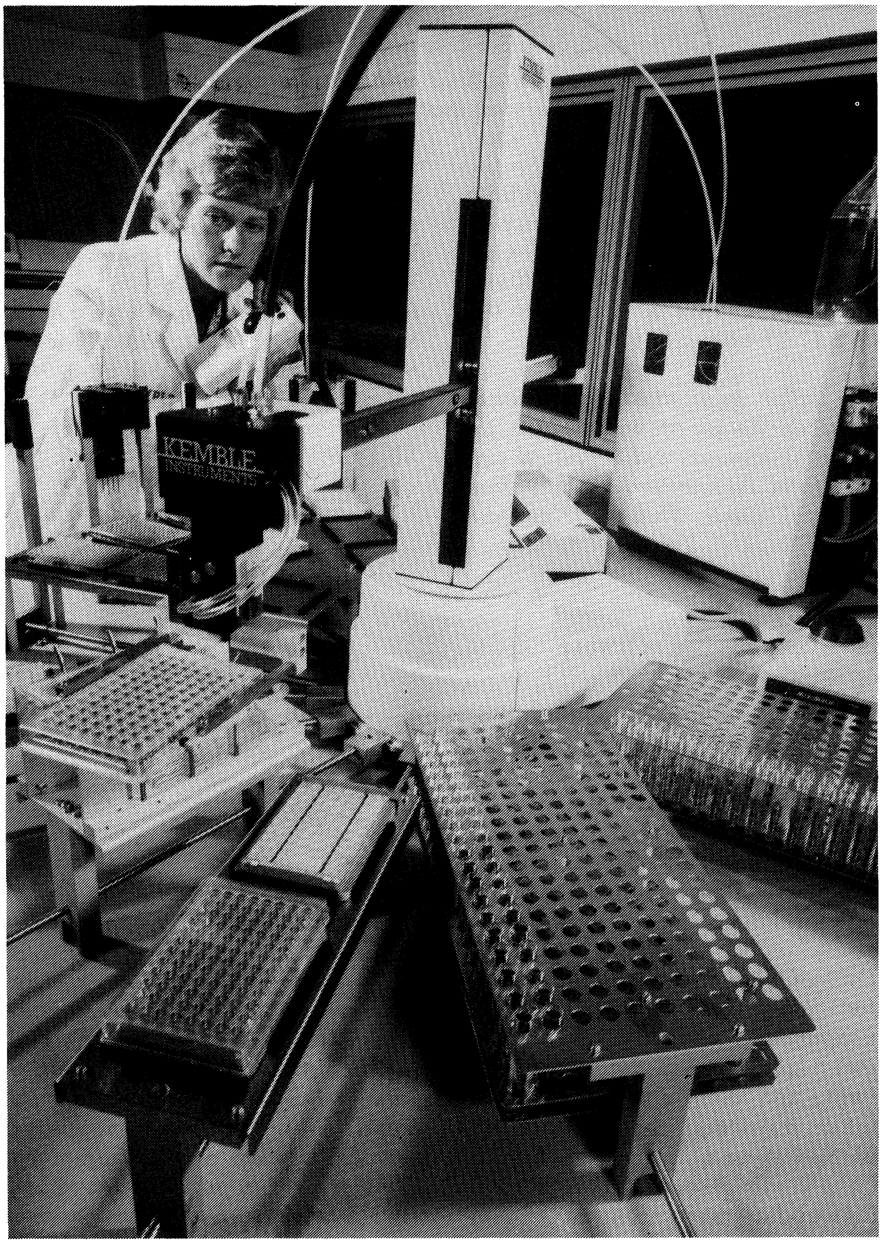

*The Autolab 500 Laboratory robot from Kemble Instrument Company.*

The different tools can be selected without human intervention, and include sampling probes, dispensing probes, tube and plate grippers, washers and lid handlers.

Sophisticated liquid handling features of the Autolab 500 include liquid level sensing, positive displacement piston pumps and dual probe sampling. The robot interacts directly with a range of peripheral equipment, such as mixers and shakers, and the software is sufficiently flexible to allow integration with many types of computer and bar code identification systems.

Simple and accurate setting up of the robot is achieved by the use of a mouse system. This facility allows finger-tip control of robot positioning and other functions. Large numbers of assay routines can be stored by the control computer, thereby allowing rapid switching between processing procedures.

Suitable environments for the Autolab 500 include virology, blood grouping and all tube or plate-based immunoassays, including RIAs, IRMAs and ELISAs.

*More information from: Kemble Instrument Co. Ltd, Marchants Way, Burgess Hill, West Sussex RH15 8QY, UK. Tel.: 0444 871187.*

### Ultrasonic slurry sampler

The USS-100 Ultrasonic Slurry Sampler for graphite furnace atomic absorption makes slurry sampling a practical analytical tool by providing full automation capabilities. The USS-100 works with the Perkin-Elmer AS-60 or AS-70 Furnace Autosamplers, and includes the parts required for installation and connection.

Some examples of slurry samples which can be successfully analysed are river sediments, coal, fly ash, plant and animal tissues and foods.

Samples are prepared as powders. Liquid is added to the sample powder, and the mixture is agitated ultrasonically to create a slurry or suspension in which the sample particles are homogeneously distributed. An aliquot of slurry is then introduced into the HGA graphite furnace for direct analysis. The USS-100 offers reduced sample preparation (and hence analysis) times with improved freedom from added contamination, easily, reliably and automatically.

*Further information from: Perkin-Elmer Ltd, Maxwell Road, Beaconsfield, Bucks HP9 1QA, UK. Tel.: 0494 676161; fax: 0494 678324.*

### Thermal desorption applications

Thermal desorption application notes are now available from Perkin-Elmer. They present details of thermal desorption-gas chromatographic analyses relevant to: environmental monitoring; occupational hygiene; and product quality control.

These applications will be of particular interest to anyone involved in these fields, either on a routine basis or as a result of recent health and safety legislation.

*For copies of the notes and details of all published thermal desorption applications and data sheets, contact: Perkin-Elmer Ltd, Maxwell Road, Beaconsfield, Buckinghamshire HP9 1QA, UK. Tel.: 0494 676161; fax: 0494 678324.*

